# Defined approaches to predict GHS and EPA classifications for ocular irritation potential of agrochemical formulations

**DOI:** 10.1080/15569527.2025.2499552

**Published:** 2025-05-15

**Authors:** Amber B. Daniel, Anna J. van der Zalm, Hans A. Raabe, Amy J. Clippinger, Neepa Y. Choksi, Emily N. Reinke, David G. Allen, Nicole C. Kleinstreuer

**Affiliations:** a Inotiv, Research Triangle Park, North Carolina, USA; b PETA Science Consortium International e.V, Stuttgart, Germany; c Institute for In Vitro Sciences, Gaithersburg, Maryland, USA; d NIH/NIEHS/DTT/NICEATM, Research Triangle Park, North Carolina, USA

**Keywords:** Eye irritation, agrochemicals, GHS, EPA, nonanimal, defined approach

## Abstract

**Introduction:**

Regulations require that agrochemicals be labeled to indicate potential harmful effects caused by exposure. The in vivo Draize rabbit eye test has historically been the standard method used to assess the eye irritation or corrosion potential of chemical substances. However, as scientific confidence has been established for certain in chemico, in vitro, and ex vivo methods developed for this purpose, regulators are increasingly accepting data from such methods in lieu of the in vivo test. Defined approaches (DAs) may also be used to derive hazard and potency predictions by applying fixed data interpretation procedures to results from multiple methods, thereby leveraging strengths of different methods. Currently, the DAs accepted by regulators to predict eye irritation or corrosion potential do not specifically list agrochemical formulations within their applicability domains.

**Methods:**

To address this gap, we conducted testing to confirm the applicability of in vitro methods to agrochemical formulations and to develop DAs to predict eye irritation hazard labeling according to the Globally Harmonized System of Classification and Labeling (GHS) and the U.S. Environmental Protection Agency (EPA) classification system. Twenty-nine formulations were tested in up to four methods: bovine corneal opacity and permeability (BCOP; OECD TG 437) including histopathology, EpiOcular Eye Irritation Test (EO; OECD TG 492), SkinEthic time-to-toxicity for liquids (TTL; OECD TG 492B), and EyeIRR-IS. We propose four DAs comprising BCOP with histopathology alone, and combined with EO, TTL, or EyeIRR-IS.

**Results and Conclusion:**

Instead of evaluating direct concordance of the four individual DAs with historical in vivo rabbit eye test data, for each formulation, we assessed orthogonal concordance of GHS and EPA classifications predicted across all five approaches. Predictions were considered orthogonally concordant when they aligned with the prediction of at least two other approaches (i.e. a majority, or at least 3 of the 5 approaches, achieved the same prediction), referred to as the ‘majority prediction.’ We also evaluated hazard labeling and PPE labeling associated with the GHS and EPA predictions, respectively. Relative to the hazard and PPE labeling associated with the majority predictions, each of the four DAs were as, or more, protective of human health than the rabbit test; hence, we conclude that these DAs can be used to predict the GHS and EPA classifications of agrochemical formulations.

## Introduction

Accidental eye exposures to chemicals can cause a range of ocular effects, depending largely on the chemical composition. Thus, it is crucial to characterize and label commercial chemical substances with information to protect people from injuries and illnesses associated with topical exposure to the eye. This information, at a minimum, includes instructions for safe handling and use, personal protective equipment (PPE) needed to protect against accidental exposure, and a warning of the potential ocular effects.

As part of the agrochemical product registration process, regulatory authorities require that companies provide information about the potential harmful effects of their products and active ingredients. Regulators use this information to evaluate the proposed hazard classification and product labeling to ensure the use directions and safety measures are adequately communicated.

In the 1940s, the in vivo Draize rabbit eye test was developed to assess the eye irritation/corrosion potential of agrochemicals [1]. While this test method has been used to characterize chemical-induced eye damage, it has documented limitations in its reliability and relevance to humans [2]. Studies have suggested that animal test responses are not necessarily relevant to humans due to interspecies differences in the anatomical and physiological mechanisms of eye irritation and corrosion [3]. Additionally, variability analyses have shown there is only an approximately 15 to 30% likelihood of obtaining the same classification result upon repeat testing for substances classified as causing mild to moderate irritation in the initial test [4]. Classifications from the in vivo rabbit eye test result from the observer’s visual assessment of ocular damage and reversibility. Therefore, interpretations of the associated apical endpoints are highly subjective, and classifications can vary between observers. Furthermore, physiological differences among individual animals may also play a role in the variable results.

Limitations of the in vivo rabbit eye test are increasingly being recognized by regulatory authorities. In January 2024, the U.S. Environmental Protection Agency (EPA) Office of Pollution Prevention and Toxics (OPPT) New Chemicals Program (NCP) issued a decision framework for identifying eye irritation or corrosion hazards for new chemical substances reviewed under the Toxic Substances Control Act, stating that NCP ‘does not encourage the prospective use of the in vivo eye irritation test using live rabbits (e.g. the Draize test),’ and instead prioritizes data from methods that use human cells or tissues or other reproducible and relevant data from in chemico, in vitro, or ex vivo methods [5].

In chemico, in vitro, and ex vivo assays (henceforth collectively referred to as ‘in vitro test methods’) have been developed for predicting eye irritation/corrosion potential, thus providing quantitative alternatives to the qualitative in vivo rabbit eye test [2]. Several of these test methods have been evaluated for their usefulness to identify potential corrosive, irritant, and/or nonirritant substances. Furthermore, some of these test methods have been adopted as Test Guidelines (TGs) by the Organization for Economic Co-operation and Development (OECD), and therefore are internationally accepted as standard methods for safety testing of chemicals.

More recently, there has been movement towards using a combination of relevant and reliable information to answer a given hazard characterization question. Defined approaches (DAs) use data generated from a prescribed set of information sources (e.g. in vitro test methods) and apply a fixed interpretation procedure to derive a prediction without needing expert judgement [6–8]. In 2022, the OECD issued TG 467 [9], which describes DAs for identifying chemicals with serious eye damage or eye irritation potential according to the classification criteria defined by the United Nations Globally Harmonized System of Classification and Labelling of Chemicals (GHS). The GHS is an international set of quality and consistency standards for communicating information about potential product health hazards [10]. However, the applicability of the DAs described in TG 467 are limited to non-surfactant neat liquids, and liquids and solids dissolved in water (the addition of a DA applicable to surfactants [11] is anticipated in 2025). Agrochemical formulations commonly contain surfactant ingredients to enhance their efficacy [12,13], thus, DAs to assess eye irritation/corrosion potential for this class of substances are needed.

Furthermore, the currently accepted OECD DAs were not developed for the classification system used by the EPA Office of Pesticide Programs (OPP). While there are some minor differences between the GHS and EPA in vivo classification criteria and respective labeling (Table 1), they are comparable four-category systems (i.e. GHS Category [Cat.] 1, 2A, 2B, and NC is like EPA Cat. I, II, III, and IV, respectively [14]). A notable difference is the labeling requirements regarding use of protective eyewear like goggles, face shields, or safety glasses. GHS requires the inclusion of hazard labeling for substances identified as GHS Cat 1, 2A, and 2B. While GHS provides examples of eye irritation/corrosion hazard statements, it allows flexibility for the manufacturer or supplier to specify appropriate PPE labeling. GHS does not require hazard labeling for substances identified as nonirritants (i.e. GHS NC). The EPA specifically requires PPE labeling as a component of the hazard labeling for substances identified as EPA Cat. I and II, but not for EPA Cat. III and IV (though the EPA may recommend inclusion of eye protection labeling for Cat. III substances, if deemed appropriate).

We previously described a study to test the eye irritation/corrosion potential of agrochemical formulations using a common set of in vitro eye irritation and corrosion test methods, which was sponsored by the National Toxicology Program (NTP) Interagency Center for the Evaluation of Alternative Toxicological Methods (NICEATM), PETA Science Consortium International e.V. (PSCI), and CropLife America [16–18]. We generated data to assess the applicability of in vitro test methods to agrochemical formulations and to support the development of DAs using one or more in vitro test methods to predict the full spectrum of eye irritation/corrosion potential of agrochemical formulations. In previous data analyses, we demonstrated the utility of DAs designed to predict the eye irritation/corrosion potential of agrochemical formulations according to the EPA classification system [18]. Since then, we have refined the previously described DAs and developed new DAs that encompass additional in vitro assays. Here we proposed four DAs and assessed their use in satisfying regulatory needs for GHS and EPA eye irritation/corrosion hazard classification and labeling of agrochemical products.

## Materials and methods

### Agrochemical formulations

Agrochemical formulations were nominated and provided for testing by CropLife America member companies, including BASF SE and BASF Corporation, Bayer CropScience, Corteva Agriscience, and Syngenta Crop Protection.

Scientists from NICEATM, PSCI, and EPA OPP selected formulations for testing based on specific inclusion criteria as follows. Only formulations with historical in vivo rabbit eye test data or ocular irritancy classification information were tested, to examine classification drivers (e.g. lesion type, persistence or reversibility of lesion). Formulations represented the full range of GHS and EPA ocular irritancy classifications derived from the in vivo rabbit eye test (Table 2).^1^ Formulations (*n* = 29) represented the most used agrochemical types, including suspension concentrates, emulsifiable concentrates, and soluble liquids, as well as one microencapsulated emulsifiable concentrate. MRIGlobal (Kansas City, MO, USA), a National Institute of Environmental Health Sciences (NIEHS) contractor, received formulation samples from the CropLife America member companies, then coded and shipped formulations to each testing laboratory.

### Study design

Full details of the testing wherein we evaluated the applicability of certain in vitro test methods to agrochemical formulations [16,17] and initial data analyses [18] have been reported. Briefly, we conducted testing in three phases. Phase 1 assessed the ability of in vitro test methods to discriminate between a small set of formulations identified as being either corrosive or nonirritating based on existing in vivo rabbit eye test data (no new in vivo testing was conducted for this study). Phase 2 expanded the set of test substances to represent the full spectrum of eye irritation/corrosion potential (based on the in vivo rabbit eye test). Based on our assessment of Phase 1 and 2 results and other factors, such as the relevance of each test method to mechanisms of human eye irritation and whether the test method was an adopted OECD TG, we selected the most promising assays to move forward for further testing in Phase 3. At the conclusion of testing, a total of 29 formulations were tested in up to five in vitro test methods^2^: 1) bovine corneal opacity and permeability (BCOP; [19]) assay (with histopathology); 2) EpiOcular^™^ (EO) Eye Irritation Test [20]; 3) SkinEthic Time-to-Toxicity approach for liquids (TTL; [21]); 4) EyeIRR-IS [22]; and 5) in vitro depth of injury (IVDoI; unpublished). We concluded that all five of these methods are applicable to agrochemical formulations, and that they may be used in the development of DAs to predict eye irritation/corrosion of agrochemical formulations [17].

For our current effort to develop DAs to predict ocular irritation/corrosion potential of agrochemical formulations, we selected only BCOP, EO, TTL, and EyeIRR-IS for inclusion in DAs. BCOP, EO, and TTL were selected because they are OECD TG assays [19–21] and thus are internationally accepted by many regulatory bodies as standard methods for safety testing of chemicals. While EyeIRR-IS is not currently described in any OECD TG, it includes several traits that indicate it may be more human relevant than the in vivo rabbit eye test, such as precise control of dosing application and termination, a 3D human tissue model, and quantitative results [2]. Additionally, for liquids, a peer-reviewed evaluation suggests it is suitable to include in a DA to replace the Draize rabbit eye test [22]. IVDoI showed some promise in preliminary evaluations [17]. However, because it has not yet been through a formal evaluation, we did not include IVDoI in DAs.

### Test methods

The selected test methods and associated prediction models are described below and summarized in Table 3.

### BCOP (with histopathology)

BCOP testing was conducted by the Institute for In Vitro Sciences, Inc. (Gaithersburg, Maryland, USA). The BCOP assay was conducted according to the method described in OECD TG 437 [19]. Briefly, bovine eyes were collected from an abattoir after slaughter for human consumption and mounted into a corneal holder. The eyes were preincubated in complete Eagle’s modified essential medium (EMEM) without phenol red. After preincubation at 32 °C, the medium was replaced with fresh medium, and initial cornea opacity was measured with an OP-KIT opacitometer. The medium on the anterior side of the corneas was then replaced with undiluted test article, negative control, or positive control. Corneas were incubated in a horizontal position for 10 min, removed, and washed with complete EMEM. The anterior chamber of the corneal holder was then refilled with complete EMEM without phenol red. Opacity was measured immediately after treatment and again after a 2 h incubation. Immediately thereafter, epithelial barrier function was assessed with a fluorescein permeability test. Sodium fluorescein solution was added to the anterior chambers and the corneas were incubated in a horizontal position for approximately 90 min. The medium was removed and transferred to a 96-well plate. Complete EMEM without phenol red was added to two wells as blank controls and the optical density at 490 nm (OD_490_) measured. The in vitro irritancy score (IVIS) for each treatment group was calculated from the corneal opacity and mean permeability values, using the equation noted in OECD TG 437 [19] and shown below.


$$IVIS\;with\;OP\text{-}KIT=mean\;opacity(read\text{-}out\;OP-KIT)\;\;\;\;\;\;\;\;\;+\left(15\times mean\;permeability\;OD_{490}\right)$$

#### Histopathology.

Histopathological evaluation of the eyes was conducted at the testing laboratory to determine depth and degree of corneal injury. Following opacity measurements, corneas were placed in tissue cassettes (equipped with a synthetic sponge to cushion the corneas) and fixed in 10% neutral buffered formalin for at least 24 h. Corneas were then embedded in paraffin for sectioning and staining with hematoxylin and eosin. Corneas were then examined for histopathological changes, beginning with the upper epithelial layer then moving down through the stromal and endothelial layers.

Previous studies have demonstrated that evaluating corneal depth of injury (DoI) within the first few hours after exposure can predict the eventual degree and duration of eye injury for most chemical classes [23–29]. Subsequent studies further developed this concept to categorize injury based on the degree and anatomical depth of cytotoxic damage [30,31]. Accordingly, the following DoI scheme, which was adapted from decision criteria presented in Redden et al. [31], was used to categorize severity of the histopathological findings (see Supplemental Figure 1):

- Minimal: damage or loss limited to the surface squamous cell layer in the epithelium; wing cell and basal cell layers remain intact.
- Mild: damage or loss extends to the wing cell layers in the epithelium; basal cell layer and basal lamina remain intact.
- Moderate: damage typically involves all layers of the epithelium and may cause keratocyte damage to the upper third to half of the stroma.
- Severe: keratocyte damage extends into the lower half of the stroma and may include damage to the endothelium.

**Figure 1.**
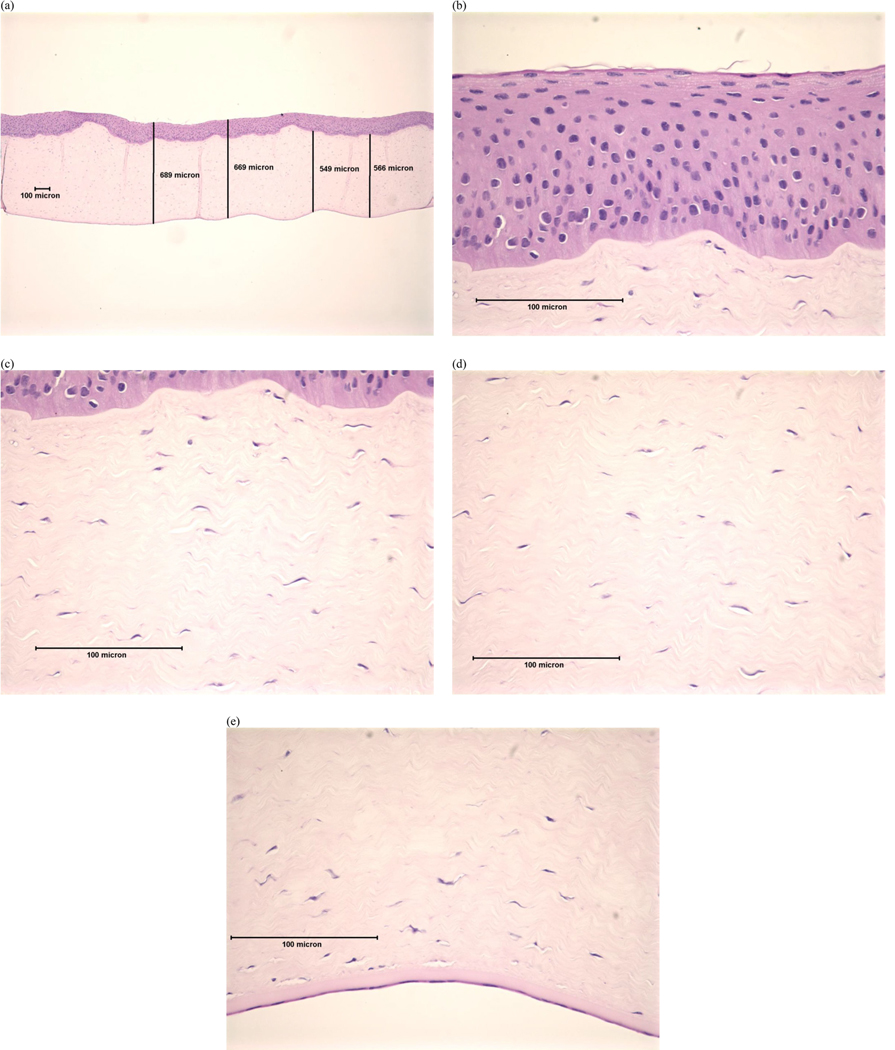
Photomicrographical images of bovine cornea treated with formulation A, shown as an example of ‘minimal’ injury determined by histopathological evaluation of (a) full thickness; (b) epithelium; (c) stroma directly beneath the anterior limiting lamina; (d) stroma at mid-depth; (e) lower stroma, Descemet’s Membrane, and endothelium. Reprinted with permission from van der Zalm et al. [18] Supplementary Material.

**Figure 2.**
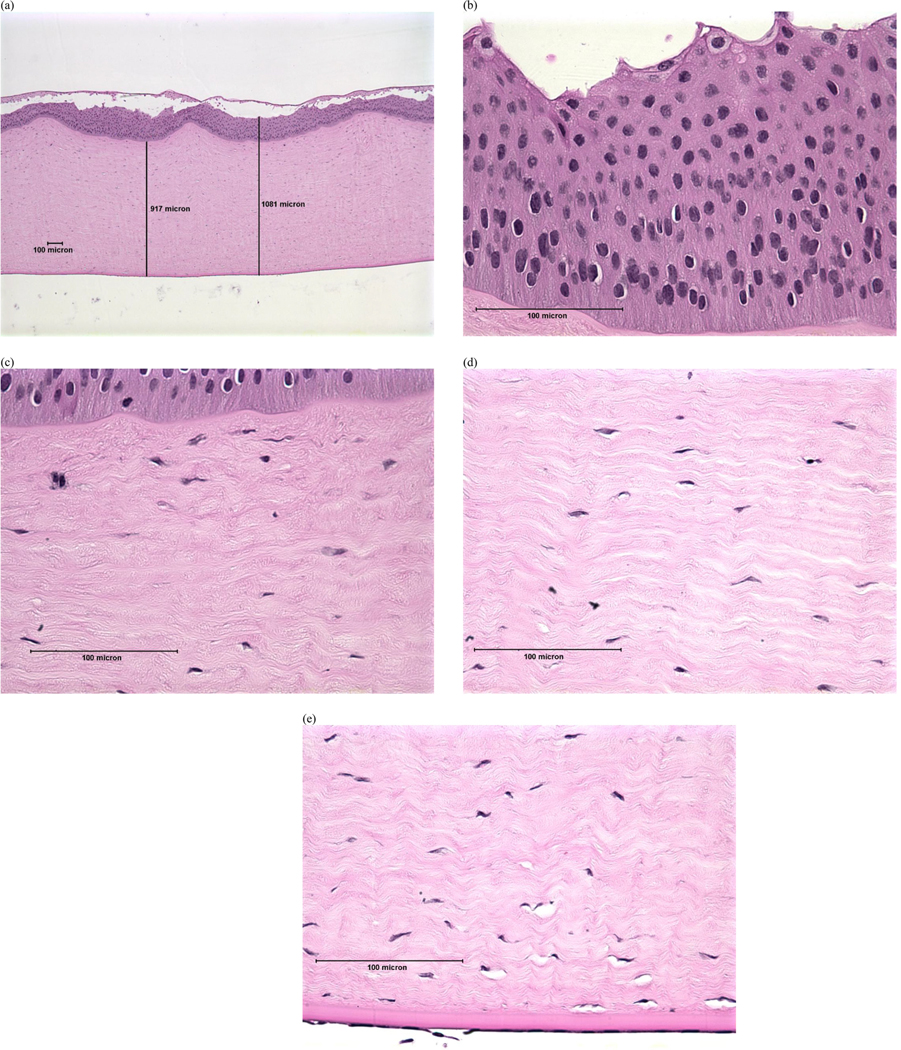
Photomicrographical images of bovine cornea treated with formulation W, shown as an example of ‘mild’ injury determined by histopathological evaluation of (a) full thickness; (b) epithelium; (c) stroma directly beneath the anterior limiting lamina; (d) stroma at mid-depth; (e) lower stroma, Descemet’s Membrane, and endothelium. Reprinted with permission from van der Zalm et al. [18] Supplementary Material.

**Figure 3.**
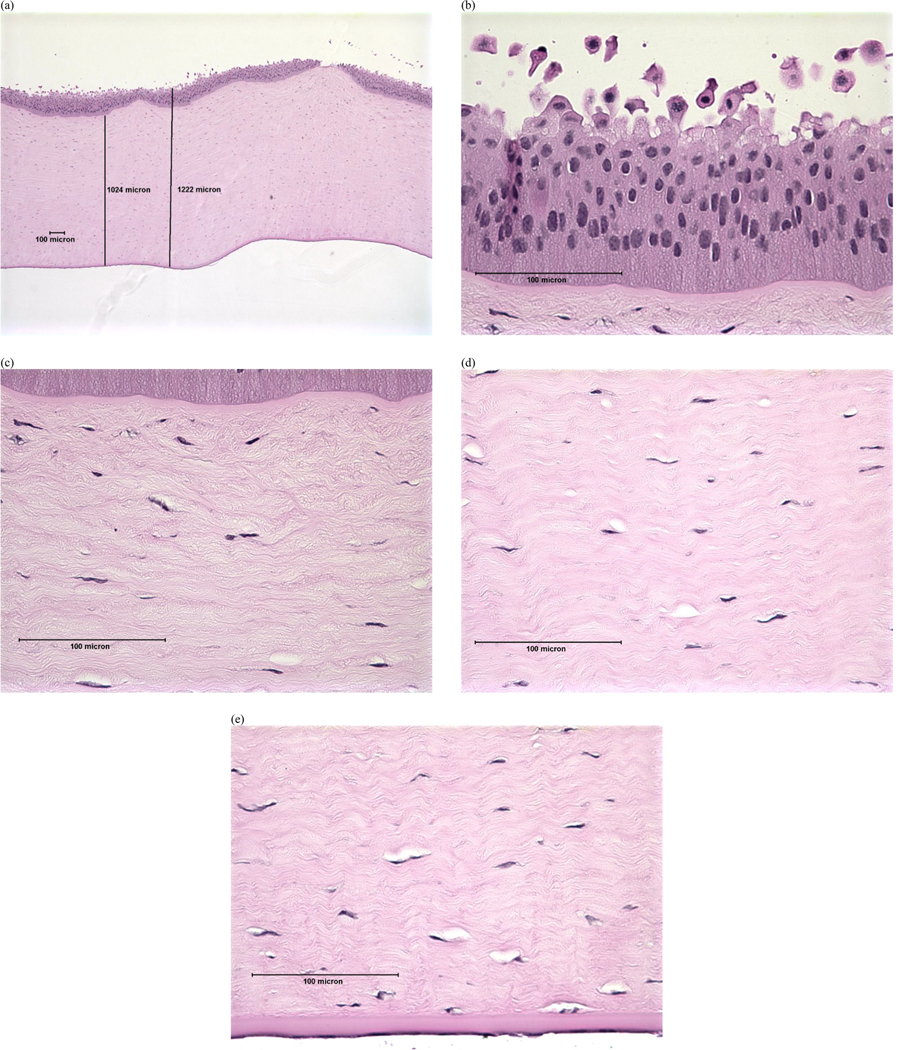
Photomicrographical images of bovine cornea treated with formulation X, shown as an example of ‘moderate’ injury determined by histopathological evaluation of (a) full thickness; (b) epithelium; (c) stroma directly beneath the anterior limiting lamina; (d) stroma at mid-depth; (e) lower stroma, Descemet’s Membrane, and endothelium. Reprinted with permission from van der Zalm et al. [18] Supplementary Material.

**Figure 4.**
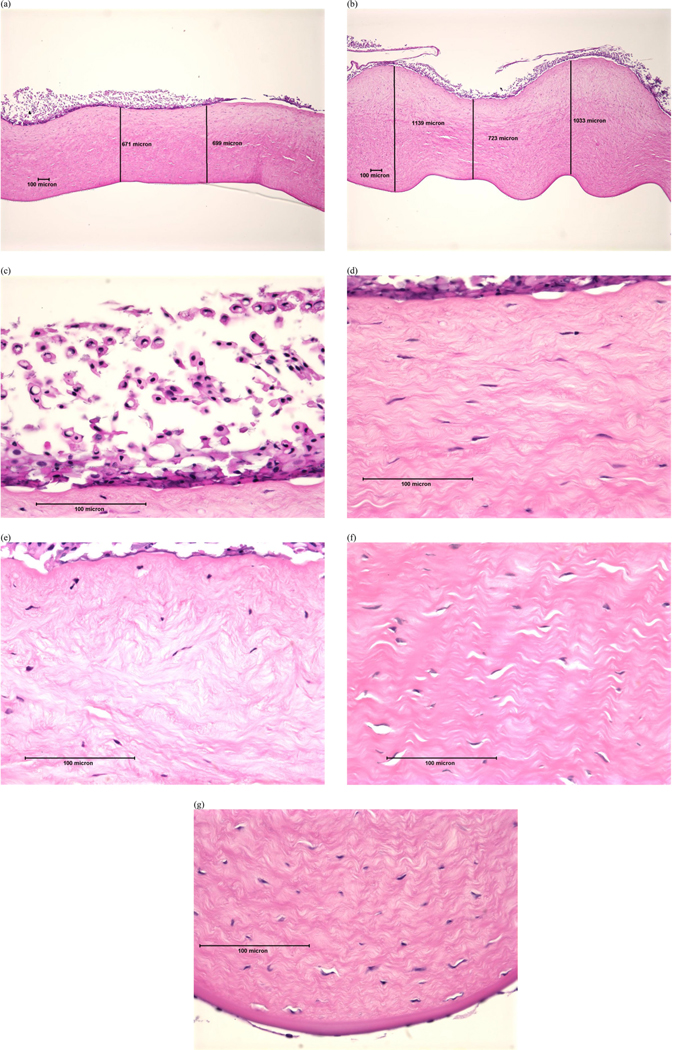
Photomicrographical images of bovine corneas treated with formulation D, shown as an example of ‘severe’ injury determined by histopathological evaluation of (a) full thickness, cornea 14; (b) full thickness, cornea 17; (c) epithelium, cornea 14; (d) stroma directly beneath the anterior limiting lamina, cornea 14; (e) stroma directly beneath the anterior limiting lamina, cornea 17; (f) stroma at mid-depth, cornea 14; (g) lower stroma, Descemet’s Membrane, and endothelium, cornea 17. Reprinted with permission from van der Zalm et al. [18] Supplementary Material.

**Figure 5.**
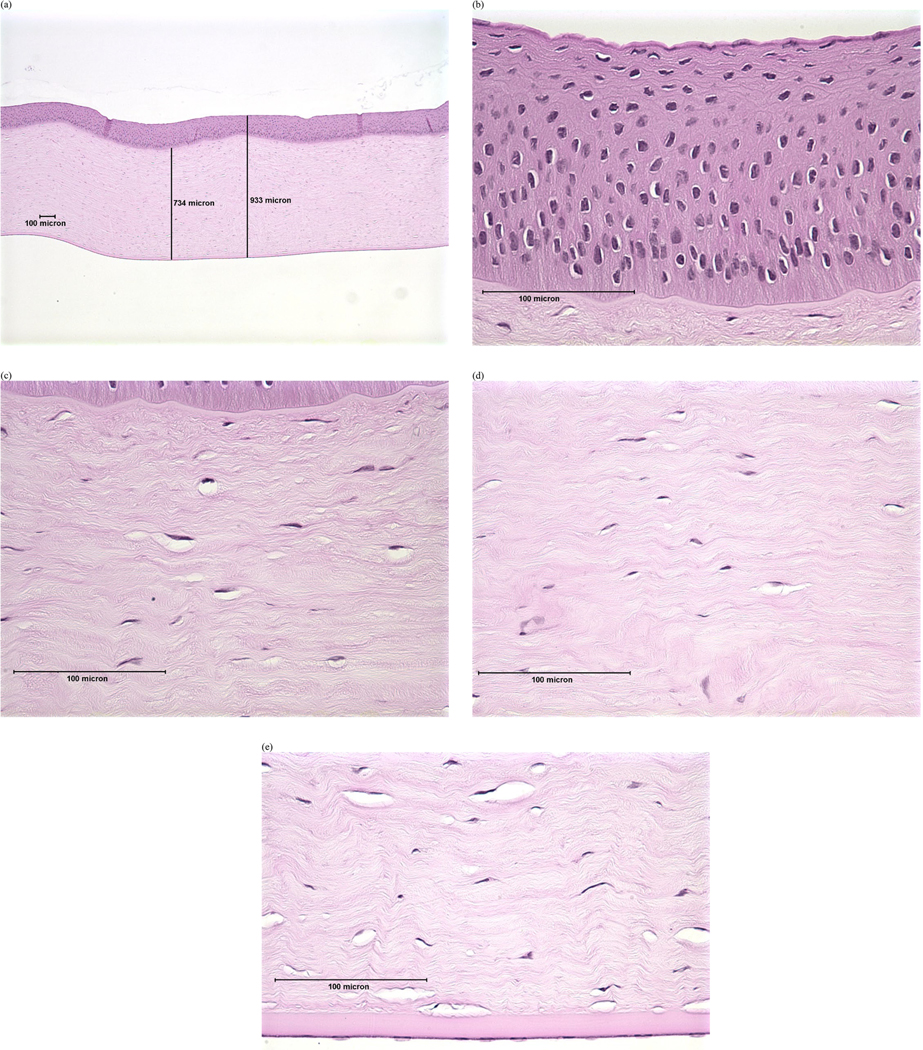
Photomicrographical images of bovine cornea treated with negative control (deionized water), as seen in histopathological evaluation of (a) full thickness; (b) epithelium; (c) stroma directly beneath the anterior limiting lamina; (d) stroma at mid-depth; (e) lower stroma, Descemet’s Membrane, and endothelium. Reprinted with permission from van der Zalm et al. [18] Supplementary Material.

**Figure 6.**
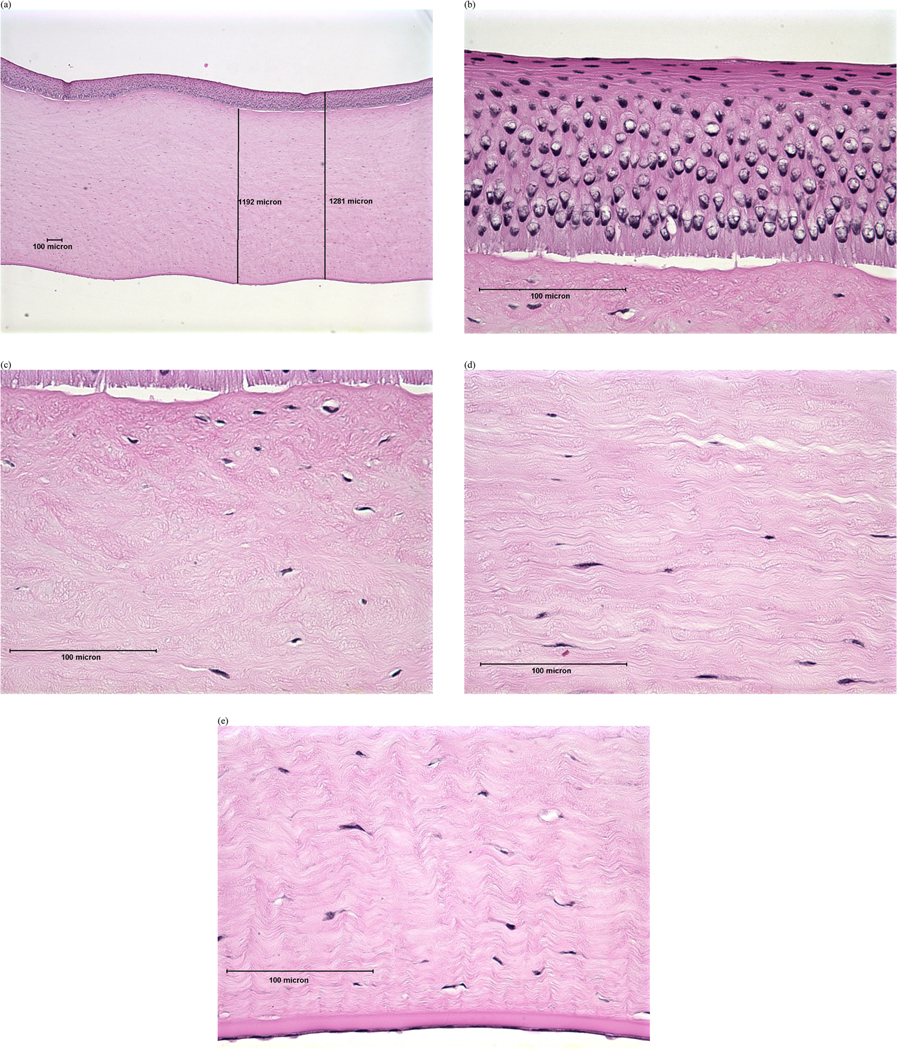
Photomicrographical images of bovine cornea treated with positive control (100% ethanol), as seen in histopathological evaluation of (a) full thickness; (b) epithelium; (c) stroma directly beneath the anterior limiting lamina; (d) stroma at mid-depth; (e) lower stroma, Descemet’s Membrane, and endothelium. Reprinted with permission from van der Zalm et al. [18] Supplementary Material.

Examples of histopathological images representing each DoI are shown in Figures 1–4. Examples of histopathological images representing negative and positive control corneas are shown in Figures 5 and 6, respectively. A detailed summary of histopathological findings is provided in the Supplementary Material for van der Zalm et al. [18].

### EO

EpiOcular^™^ (EO) testing was conducted by MatTek (Bratislava, Slovak Republic), according to the assay method described in OECD TG 492 [20]. Briefly, test articles or controls were applied to EO reconstructed human cornea-like epithelium tissues (RhCE) and incubated for 30 min. Inserts containing the tissues were removed from the wells and rinsed. The inserts were then cultured for a 2 h incubation. Next, the inserts were transferred into 24-well plates and incubated with 3–(4,5-dimethylthiazo-2-yl)-2,5-diphenyl-tetrazolium bromide (MTT) dye for 180 ± 15 min, rinsed with Dulbecco’s phosphate-buffered saline (PBS), and incubated overnight with isopropanol. The next day, the plates were placed on an orbital shaker for 2–3 h at room temperature. MTT solution was then placed on a 96-well plate and absorbance measured at 570 nm to determine tissue viability.

### TTL

TTL testing was conducted by Episkin (Lyon, France), according to the method described in OECD TG 492B [21]. Briefly, formulations were applied topically to maturation day 5 RhCE tissues for exposure durations of 5, 16, and 120 min, and then rinsed with PBS. Formulations were tested neat for 5-min exposures and formulations were diluted 20% (w/v) with water for 16- and 120-min exposures. After rinsing, tissues were placed in fresh medium at room temperature for 10 min. The lab used the MTT assay to quantitatively determine percent viability of the tissues exposed to the test articles, relative to that of tissue treated with the negative control, for each of the three exposure times.

### EyeIRR-IS

EyeIRR-IS testing was conducted by ImmunoSearch (Grasse, France), according to the method described in Cottrez et al. [22]. Briefly, each formulation was tested neat and also diluted at 30% with PBS. The RhCE tissue surface was moistened with PBS and incubated for 10 min at 37 °C. An epithelium was then topically treated with 50 ± 2 μL of the test formulation preparation (corresponding to 100 μL/cm^2^) and incubated 10 min at room temperature. The test article was then gently rinsed from the tissue by spraying sterile PBS against the cell culture insert wall, not directly on the tissue. The tissues still resting on their insert support were then soaked in 5 ml of culture medium for 30 min to ensure complete removal of any remaining test article. The rinsing medium was removed, and fresh culture medium was added. Tissues were then incubated for 6 h. Total RNA was extracted, and quantitative reverse transcription-polymerase chain reaction was performed for gene expression analysis of 10 genes. An algorithm based on gene expression modulation was used to calculate a liquid irritation index (LII), which is the quantitative representation of tissue damage between 0 and 20 that forms the basis of the EyeIRR-IS prediction model.

### In vivo Draize rabbit eye test

No prospective in vivo testing was conducted for this evaluation. CropLife America member companies provided historical in vivo rabbit eye test data and study details required to derive ocular irritation classifications for the test formulations, such as number of rabbits tested and number of animals driving the classification. We only included data from studies conducted according to the methodology described in OECD TG 405 [32].

### Defined approaches

**Figure 7.**
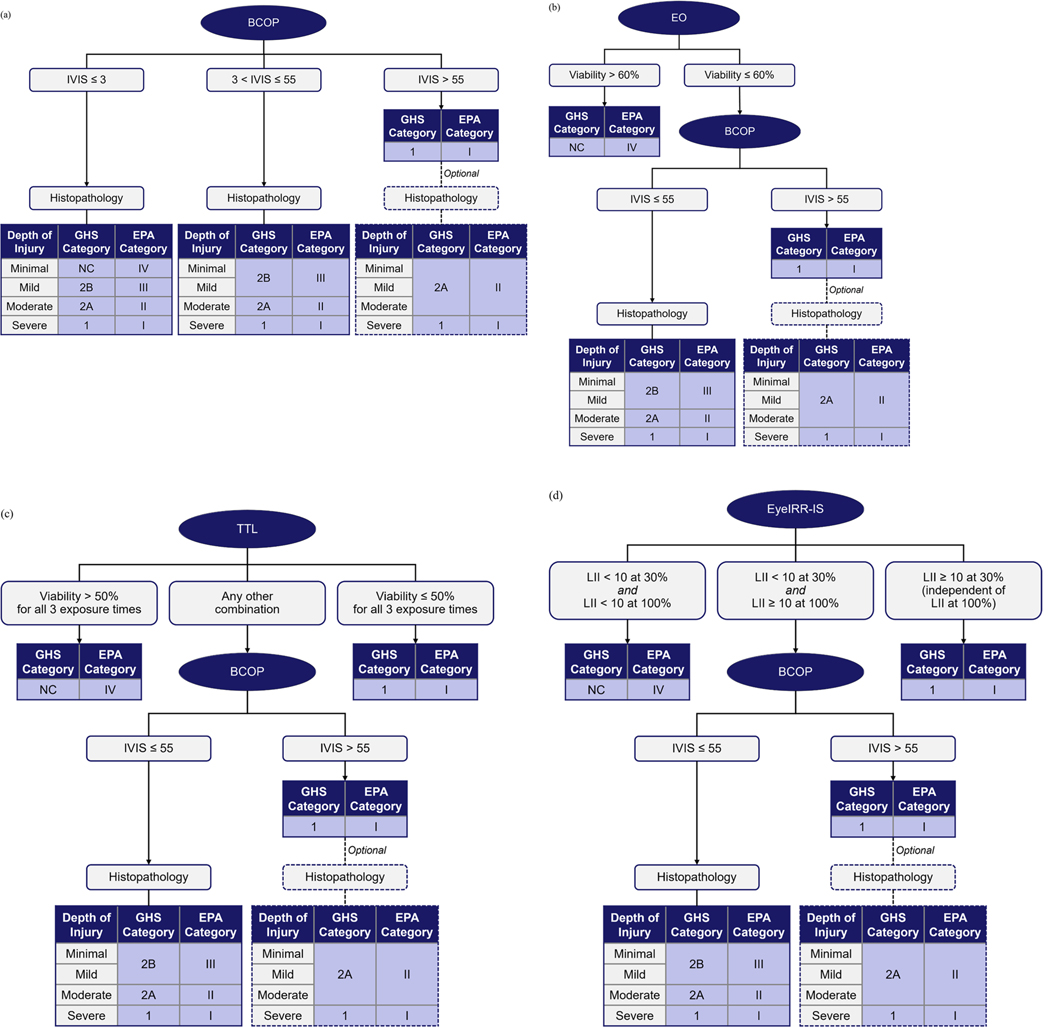
Schematics demonstrate the classification flow chart for GHS and EPA hazard classification of eye irritation/corrosion potential of agrochemical formulations using defined approaches (a) DA-BCOP+; (b) DA-EO+; (c) DA-TTL+; (d) DA-EyeIRR-IS+.

Expanding on our previous data analyses [16–18], we have proposed four DAs to predict GHS and EPA hazard classifications for eye irritation/corrosion potential: BCOP with histopathology alone (‘DA-BCOP+’); and EO, TTL, or EyeIRR-IS combined with BCOP with histopathology: (‘DA-EO+,’ ‘DA-TTL+,’ and ‘DA-EyeIRR-IS+,’ respectively). The stand-alone methods included in the DAs (i.e. BCOP, EO, TTL, and EyeIRR-IS) do not all include EPA classification criteria in their respective prediction models. Because of this and given the similarity of the two classification systems (Table 1), we considered the classification criteria of EPA Cat. I, II, III, and IV coequal with that of GHS Cat. 1, 2A, 2B, and NC, respectively, for the development of these DAs. The proposed DAs are shown in Figure 7 and described in the sections below.

### DA-BCOP+

DA-BCOP+ is adapted from a previously described DA that predicts the eye irritation/corrosion potential of agrochemical formulations according to the EPA classification system [18] but is refined to offer increased protection to human health and demonstrate applicability to both the GHS and EPA classification systems. DA-BCOP+ comprises a single assay, but with multiple integrated endpoints: BCOP (with histopathology). OECD endorses BCOP as a scientifically valid assay for (1) identifying chemicals and mixtures that may induce serious eye damage (i.e. GHS Cat. 1) and (2) those not classified for eye irritation or serious eye damage (i.e. GHS NC) based on the calculated IVIS [19]. Including histopathology in the BCOP may reduce underprediction of certain agrochemical formulations. Specifically, the IVIS calculated for formulations Q, S, and Y were ≤ 3 (corresponding to GHS NC/EPA Cat. IV if predictions were based on IVIS alone), while the histopathology findings indicated potential for mild (GHS Cat. 2B/EPA Cat. III; formulations S and Y) or moderate (GHS Cat. 2A/EPA Cat. II; formulation Q) eye irritation. Therefore, DA-BCOP+ (Figure 7a) includes histopathological DoI analysis when IVIS ≤ 3, to confirm GHS NC classification or to upgrade to a more severe classification. For formulations where 3 < IVIS ≤ 55 (corresponding to ‘no stand-alone prediction can be made’ based on the original BCOP prediction model), DA-BCOP+ includes histopathological DoI analysis to determine the GHS and EPA classification. It should be noted that 3 < IVIS ≤ 55 implies some potential for irritation, and therefore GHS NC/EPA Cat.

IV is not achievable even if histopathological findings suggest ‘minimal’ injury. Consistent with OECD TG 437, DA-BCOP+ classifies a formulation as GHS Cat. 1/EPA Cat. I if IVIS > 55. However, DA-BCOP+ allows a classification downgrade to GHS Cat. 2A/EPA Cat. II if histopathological DoI analysis findings indicate that injuries would be less severe.

### DA-EO+

DA-EO+ is adapted from a previously described DA that predicts the eye irritation/corrosion potential of agrochemical formulations according to the EPA classification system [18] but is proposed here as being applicable to both the GHS and EPA classification systems. DA-EO+ (Figure 7b) begins with the EO assay, based on cell viability, to identify substances not classified for eye irritation or serious eye damage. If viability is > 60%, the formulation is GHS NC/EPA Cat. IV and testing stops. If viability is ≤ 60%, BCOP is used to assess potential delayed cytotoxic effects and degree of eye damage. Where IVIS ≤ 55, histopathological DoI analysis is used to determine GHS and EPA classification, or a formulation is classified GHS Cat. 1/EPA Cat. I if IVIS > 55. As with DA-BCOP+, if histopathological DoI analysis findings indicate that injuries are less severe, the GHS Cat. 1/EPA Cat. I classification may be downgraded to GHS Cat. 2A/EPA Cat. II.

### DA-TTL+

DA-TTL+ (Figure 7c) begins with cell viability measurement in the TTL assay to identify substances not classified for eye irritation or serious eye damage. If viability > 50% for all three exposure times, the formulation is GHS NC/EPA Cat. IV and testing stops. If viability is ≤ 50% for all three exposure times, the formulation is GHS Cat. 1/EPA Cat. I and testing stops. If any other combination of results is achieved, BCOP is used to assess the degree of eye damage. As with the previous DAs, for formulations where IVIS ≤ 55, DA-TTL+ includes histopathological DoI analysis to determine the appropriate GHS and EPA classification, or classifies a formulation as GHS Cat. 1/EPA Cat. I if IVIS > 55. As with DA-EO+, if histopathological DoI analysis finds that the injury is less severe, the classification may be downgraded to GHS Cat. 2A/EPA Cat. II.

### DA-EyeIRR-IS+

DA-EyeIRR-IS+ (Figure 7d) begins with the EyeIRR-IS assay to identify substances not classified for eye irritation or serious eye damage based on calculated LII. If LII < 10 at both the 30% and 100% concentrations, the formulation is GHS NC/EPA Cat. IV and testing stops. If LII ≥ 10 at 30% (independently of the LII value obtained at 100%), the formulation is GHS Cat. 1/EPA Cat. I and testing stops. If LII < 10 at 30% and LII ≥ 10 at 100%, BCOP is used to assess the degree of eye damage. DA-EyeIRR-IS+ uses the same IVIS thresholds combined with histopathological DoI analysis to determine the GHS and EPA classification as DA-EO+ and DA-TTL+.

### Data analysis

Given the limitations and low reliability of the in vivo rabbit eye test [2], it was not appropriate to assess performance of the DAs based solely on concordance of predictions with that of the in vivo data. Instead, to overcome the limitations of any one approach, we conducted orthogonal concordance analyses to evaluate the performance of the DAs.

For each formulation, we used in vitro testing data to apply the four DAs. We then orthogonally compared the GHS and EPA classifications predicted by the DAs and by the historical rabbit eye test data^3^ against each other (i.e. for each formulation, concordance was evaluated based on agreement across the five approaches). Predictions were considered orthogonally concordant when they aligned with the prediction of at least two other approaches (i.e. a majority, or at least 3 of the 5 approaches, achieved the same prediction). Predictions were considered orthogonally discordant when they misaligned with the majority prediction. We also evaluated hazard labeling and PPE labeling associated with the GHS and EPA predictions, respectively.

## Results

Results from testing of the methods included in the DAs (i.e. BCOP with histopathology, EO, TTL, and EyeIRR-IS in vitro assays) are summarized in Supplemental Table 1. Full in vitro test results and historical in vivo rabbit eye test data are reported in the Supplementary Material for Choksi et al. [16] and Daniel et al. [17]. GHS and EPA classifications predicted by the DAs are presented in Tables 4 and 5, respectively. Additionally, determinants of the classifications predicted by the DAs (i.e. the step of the flow chart in which the prediction is determined, and testing stops) are shown in Supplemental Table 2.

### GHS

Results of the orthogonal analysis of GHS classifications predicted by DAs and historical in vivo data are presented in Table 4. The option to potentially downgrade a classification of GHS Cat. 1 to Cat. 2A (based on histopathological DoI analysis indicating less severe injury following IVIS > 55), which is offered in all the DAs, is not reflected in these results.

When GHS classifications and associated hazard labeling predicted by each of the DAs and historical in vivo data were orthogonally evaluated, there was majority alignment across approaches (i.e. at least 3 of 5 approaches) for 97% (28/29) of formulations. Of these, there was alignment across three, four, and all five approaches for 29% (8/28), 25% (7/28), and 46% (13/28) formulations, respectively. Data were insufficient to determine alignment across approaches for Formulation AB.

Orthogonal discordance among the DAs was noted as follows:

- DA-BCOP+: 4 formulations had orthogonally discordant results. Relative to that of the majority predictions, the GHS requirement for hazard labeling was overprotective for 50% (2/4; Formulations T and Z) and underprotective for 50% (2/4; Formulations K and AA).
- DA-EO+: 2 formulations had orthogonally discordant results. Relative to that of the majority predictions, the GHS requirement for hazard labeling was overprotective for 100% (2/2; Formulations L and O).
- DA-TTL+: 4 formulations had orthogonally discordant results. Relative to that of the majority predictions, the GHS requirement for hazard labeling was maintained for 25% (1/4; Formulation R) and overprotective for 75% (3/4; Formulations L, O, and T).
- DA-EyeIRR-IS+: 5 formulations had orthogonally discordant results. Relative to that of the majority predictions, the GHS requirement for hazard labeling was maintained for 80% (4/5; Formulations E, R, U, and x) and underprotective for 20% (1/5; Formulation AC); there were no formulations for which DA-EyeIRR-IS+ produced overprotective results.

There were 8 formulations for which the results based on historical in vivo data were orthogonally discordant. Of these, the GHS requirement for hazard labeling was maintained for 62.5% (5/8; Formulations E, K, V, Y, and AA). Hazard labeling was underprotective for the remaining 37.5% (3/8; Formulations Q, W, and AC). There were no formulations for which the historical in vivo data produced overprotective results.

### EPA

Results of the orthogonal analysis of EPA classifications predicted by DAs and historical in vivo data are presented in Table 5. The option to potentially downgrade a classification of EPA Cat. I to Cat. II (based on histopathological DoI analysis indicating less severe injury following IVIS > 55), which is offered in all the DAs, is not reflected in these results.

When EPA classifications and associated PPE labeling predicted by each of the DAs and historical in vivo data were orthogonally evaluated, there was majority alignment across approaches (i.e. at least 3 of 5 approaches) for 97% (28/29) of formulations. Of these, there was alignment across three, four, and all five approaches for 29% (8/28), 18% (5/28), and 54% (15/28) formulations, respectively. Data were insufficient to determine alignment across approaches for Formulation AB.

Orthogonal discordance among the DAs was noted as follows:

- DA-BCOP+: 4 formulations had orthogonally discordant results. Relative to that of the majority predictions, the EPA requirement for PPE labeling was maintained for 100% (4/4; Formulations K, L, Z, and AA).
- DA-EO+: 2 formulations had orthogonally discordant results. Relative to that of the majority predictions, the EPA requirement for PPE labeling was maintained for 100% (2/2; Formulations O and T).
- DA-TTL+: 2 formulations had orthogonally discordant results. Relative to that of the majority predictions, the EPA requirement for PPE labeling was maintained for 100% (2/2; Formulations O and R).
- DA-EyeIRR-IS+: 7 formulations had orthogonally discordant results. Relative to that of the majority predictions, the EPA requirement for PPE labeling was maintained for 71% (5/7; Formulations L, R, T, x, and AC) and overprotective for 29% (2/7; Formulations E and U).

There were 6 formulations for which the results based on historical in vivo data were orthogonally discordant. Relative to that of the majority predictions, the EPA requirement for PPE labeling was maintained for 17% (1/6; Formulation Z) and overprotective for 67% (4/6; Formulations E, K, Y, and AA). PPE labeling was underprotective for the remaining 17% (1/6; Formulation V).

## Discussion

The in vivo rabbit eye test has long been a subject of controversy based on variability, subjectivity, and animal welfare concerns (documented as early as 1971 by Weil and Scala [33]). While increasing confirmation of these limitations (e.g. [2,4,34,35]) is recognized in some regulatory frameworks [5], other regulators continue to rely on the in vivo rabbit eye test due to the view that in vitro results should align with traditional in vivo test results [36]. A lack of alignment between in vitro and in vivo results is particularly evident for substances identified as mild or moderate irritants by the in vivo test. Notably, this is the same level of irritation where the in vivo test cannot reliably reproduce itself, with approximately 15 to 30% concordance among repeat tests on the same substances [4].

Recognizing the valid concerns raised in these works, our current study employed a strategy to assess the DAs performance using orthogonal concordance analysis, instead of directly comparing solely with the in vivo rabbit eye test data. The results further emphasize that the in vivo rabbit eye test is not always reliable and, in some cases, may result in hazard labeling that is underprotective of human health.

Agrochemical companies should determine which DA to use for testing eye irritation/corrosion potential of a new agrochemical formulation on a case-by-case basis. Knowledge of a formulation’s composition can be an important consideration. For example, if a formulation is expected to be nonirritating based on physicochemical properties, read-across studies, or historical data, DA-EO+ may provide the most efficient pathway to GHS NC/EPA Cat. IV classification. If a formulation is expected to be irritating, DA-EyeIRR-IS+ may provide the most insight on reversibility of corneal injury since the EyeIRR-IS assay measures expression of genes involved in the wound healing process [22]. Previous studies have proposed that histopathological DoI analysis, which is included in all the DAs, may be useful in characterizing the reversibility of corneal effects [26,37]. Other practical considerations for determining which DA to use include availability of tissues, reagents, and/or equipment required to conduct testing.

Each of the stand-alone assays selected for inclusion in the DAs to predict GHS or EPA classification for ocular irritation/corrosion potential of agrochemical formulations have been characterized as being as or more human relevant than the in vivo rabbit eye test [2]. Integrating multiple methods with different strengths in DAs can be useful in overcoming any limitations of an individual component method. For example, DA-EO+ leverages the use of human-relevant cells in EO testing, and BCOP (if necessary, based on the outcome of EO testing) adds a full-thickness corneal morphology aspect which provides the ability to classify potential irritants and differentiate between levels of severity.

For both the GHS and EPA classification systems, a majority alignment was observed across approaches (i.e. at least 3 of 5 approaches) for all formulations except Formulation AB. Notably, this is the only formulation which was not tested in all in vitro test methods due to insufficient volume of the donated sample. Additionally, for both classification systems, each of the DAs performed better than the historical in vivo data with respect to number of formulations with underpredicted hazard labeling or PPE labeling.

The DAs were developed with the objective of protecting human health and may therefore be conservative (i.e. tending toward overprotection) in some cases. Of greater concern are approaches that produce a classification and associated hazard labeling that is underprotective of potential eye irritation or corrosion relative to the majority prediction. It was not feasible to conduct a thorough investigation of possible causes for orthogonal discordance among predictions since the donating agrochemical companies did not provide proprietary information about composition of the tested formulations. However, we were able to make some relative comparisons across approaches.

Based on results of the GHS analysis, the historical in vivo data produced three orthogonally discordant classifications that would remove a requirement for hazard labeling (i.e. underprotective compared with the majority prediction). DA-BCOP+ produced two such classifications, and DA-EyeIRR-IS+ produced one. Based on results of the EPA analysis, only the historical in vivo data produced an orthogonally discordant classification that would remove a requirement for PPE in comparison with the majority prediction. Previous work has demonstrated the utility of similar DAs designed to predict the eye irritation/corrosion potential of agrochemical formulations [18], and the results of our orthogonal analyses further support high confidence in the use of DAs for this purpose.

The DAs proposed in this work, namely the DA-BCOP+, DA-EO+, DA-TTL+, and DA-EyeIRR-IS+, were all developed using well characterized, reproducible assays that use precise, quantitative systems to measure exposure and effects. We conclude that DA-BCOP+, DA-EO+, DA-TTL+, and DA-EyeIRR-IS+ are equally or more protective of human health than the in vivo rabbit eye test, and that these DAs are applicable to both the GHS and the EPA classification systems. This translates to these DAs being as good as or better than the in vivo rabbit eye test for predicting eye irritation or corrosion potential of agrochemical formulations in humans. Therefore, these DAs present an opportunity to fully replace the use of the in vivo rabbit eye test for determining GHS and EPA hazard classification and labeling of agrochemical formulations in regulatory frameworks.

## Supplementary Material

Supplementary Materials

## Figures and Tables

**Table 1.** Summarized comparison^a^ of GHS and EPA classification and labeling systems.

GHS In Vivo Classification Criteria, and Required Signal Words, Pictograms, and Hazard Statements	EPA OPP In Vivo Classification Criteria, and Required Signal Words, Symbols, Hazard Statements, and PPE Labeling^b^

**Category 1** Animal test data indicate that the substance produces either: (1) in at least one animal, effects on the cornea, iris or conjunctiva that are not expected to reverse or have not reversed within 21 days; or (2) in at least two of three tested animals, a positive response of corneal opacity ≥ 3 and/or iritis > 1.5 (calculated as the mean scores following grading at 24, 48, and 72 hours after instillation). • DANGER • Corrosion symbol in diamond • Causes severe eye damage	**Category I** Corrosive (irreversible destruction of ocular tissue) or corneal involvement or irritation persisting for more than 21 days. • DANGER • No symbol • Corrosive. Causes irreversible eye damage • Appropriate protective eyewear (such as goggles, face shield, or safety glasses)
**Category 2A** Animal test data indicate that the substance produces a positive response in at least two of three tested animals of: corneal opacity ≥ 1, iritis ≥ 1, conjunctival redness ≥ 2, or conjunctival chemosis ≥ 2 (calculated as the mean scores following grading at 24, 48, and 72 hours) and the effects are reversible within 21 days. • WARNING • Exclamation mark in diamond • Causes severe eye irritation	**Category II** Corneal involvement or irritation (i.e. corneal opacity or iritis ≥ 1, or conjunctival chemosis or conjunctival redness ≥ 2) clearing^c^ in 8–21 days. • WARNING • No symbol • Causes substantial but temporary eye injury • Appropriate protective eyewear (such as goggles, face shield, or safety glasses)
**Category 2B** The substance meets classification criteria for 2A, and such effects are reversible within 7 days. • WARNING • No pictogram • Causes eye irritation	**Category III** Corneal involvement or irritation (i.e. corneal opacity or iritis ≥ 1, or conjunctival chemosis or conjunctival redness ≥ 2) clearing^c^ in 7 days or less. • CAUTION • No symbol • Causes moderate eye irritation • No PPE labeling required. Registrant may choose to specify protective eyewear, if appropriate
**Not Classified** No effects are produced, or minimal effects observed that do not lead to classification. • No hazard labeling required	**Category IV** Minimal effects (i.e. corneal opacity or iritis ≥ 1, or conjunctival chemosis ≥ 2) clearing^c^ in less than 24 hours. • No signal word, symbol, hazard statement, or PPE labeling required. Registrant may choose to use Category III statement

a Chemical Hazard Classification and Labeling: Comparison of OPP Requirements and the GHS [14].

b Label Review Manual – Chapter 7: Precautionary Statements [15].

c Corneal opacity or iritis scores of 0 and conjunctival chemosis or conjunctival redness scores of ≤ 1 are considered cleared.

Abbreviations: EPA OPP = U.S. Environmental Protection Agency Office of Pesticide Programs; GHS = Globally Harmonized System of Classification and Labeling of Chemicals (GHS); PPE = personal protective equipment.

**Table 2.** Tested agrochemical formulations.

Formulation code	Formulation type	Historical in vivo GHS classification	Historical in vivo EPA classification

A	EC/ME	NC	IV
B	SC	NC	IV
C	SC	NC	IV
D	EC	1	I
E	EC	1	I
F	SL	1	I
G	EC	1	I
H	SL	1	I
I	SL	1	I
J	EC	1	I
K	SL	2A	II
L	EC	NC	III
M	SL	NC	IV
N	SC	NC	IV
O	SL	NC	IV
P	SC	NC	IV
Q	SL	NC	II
R	SL	2A	II
S	SL	2B	III
T	SC	NC	III
U	EC	2A	II
V	SL	2B	III
W	SL	NC	III
X	EC	2A	II
Y	EC	2A	II
Z	EC	NC	III
AA	EC	2A	II
AB	EC	2B	III
AC	EC	NC	III

Abbreviations: EC = emulsifiable concentrate; ME = microencapsulated; SC = suspension concentrate; SL = soluble liquid.

**Table 3.** In vitro test methods/protocols and classification criteria for GHS ocular irritancy categories.

Test method/protocol	GHS classification				NPCBM

	NC	2B	2A	1	

BCOP^a^	IVIS ≤ 3	NA	NA	IVIS > 55	3 < IVIS ≤ 55
EO^b^	Viability > 60%	NA	NA	NA	Viability ≤ 60%
TTL^c,d^	Viability > 50% for all three exposure times	Any other combination		Viability ≤ 50% for all three exposure times	NA
EyeIRR-IS^d^	LII < 10 at 30% and LII < 10 at 100%	LII < 10 at 30% and LII ≥ 10 at 100%		LII ≥ 10 at 30% (independently of the LII value obtained at 100%)	NA

a OECD Test Guideline 437 [19].

b OECD Test Guideline 492 [20].

c OECD Test Guideline 492B [21].

d Prediction model does not distinguish GHS 2A/2B sub-categories.

Abbreviations: BCOP = bovine corneal opacity and permeability; DoI = stromal depth of injury; histo = histopathology; EO = EpiOcular; IVDoI = in vitro depth of injury; IVIS = in vitro irritancy score; LII = liquid irritation index; meta = metabolic; NA = not applicable; NC = not classified; neg = negative; NPCBM = no stand-alone prediction can be made; pos = positive; TTL = SkinEthic Time-to-Toxicity approach for liquids.

**Table 4.** Orthogonal concordance of GHS classifications predicted by DAs and historical in vivo data.

Formulation code	DA-BCOP+	DA-EO+	DA-TTL+	DA-EyeIRR-IS+	Historical in vivo	Majority prediction

A	NC	NC	NC	NC	NC	NC
B	NC	NC	NC	NC	NC	NC
C	NC	NC	NC	NC	NC	NC
D	1	1	1	1	1	1
E	2B	2B	2B	1	1	2B
F	1	1	1	1	1	1
G	1	1	1	1	1	1
H	1	1	1	1	1	1
I	1	1	1	1	1	1
J	1	1	1	1	1	1
K	NC	2B	2B	2B	2A	2B
L	NC	2B	2B	NC	NC	NC
M	NC	NC	NC	NC	NC	NC
N	NC	NC	NC	NC	NC	NC
O	NC	2B	2B	NC	NC	NC
P	NC	NC	NC	NC	NC	NC
Q	2A^a^	2A	2A	2A	NC	2A
R	2A	2A	1	1	2A	2A
S	2B^a^	2B	2B	2B	2B	2B
T	2B^a^	NC	2B	NC	NC	NC
U	2A	2A	2A	1	2A	2A
V	1^b^	1^b^	1^b^	1^b^	2B	1
W	2B	2B	2B	2B	NC	2B
X	2A	2A	2A	1	2A	2A
Y	2B^a^	2B	2B	2B	2A	2B
Z	2B	NC	NC	NC	NC	NC
AA	NC	2B	2B	2B	2A	2B
AB	2A	2A	Not tested	Not tested	2B	None
AC	2B	2B	2B	NC	NC	2B
**Orthogonally concordant**	**24/28; 86%**	**26/28; 93%**	**24/28; 86%**	**23/28; 82%**	**20/28; 71%**	
**Orthogonally discordant**	**4/28; 14%**	**2/28; 7%**	**4/28; 14%**	**5/28; 18%**	**8/28; 29%**	
Hazard labeling maintained^c^	0	0	1	4	5	
Hazard labeling overprotective^c^	2	2	3	0	0	
Hazard labeling underprotective^c^	2	0	0	1	3	

a IVIS < 3, but histopathology DoI analysis led to a more severe classification.

b Optional histopathology DoI analysis would lead to a less severe classification (i.e. GHS Cat. 2A).

c Relative to that of the majority prediction.

Green = orthogonally concordant with majority prediction (i.e. aligned across three or more approaches).

Yellow = orthogonally discordant with majority prediction, but the requirement for hazard labeling is maintained.

Blue = orthogonally discordant with majority prediction that would drive the inclusion of hazard labeling (overprotective).

Red = orthogonal discordance with majority prediction that would result in hazard labeling not being required (underprotective).

No fill = no majority alignment across approaches.

Abbreviations: NC = not classified.

**Table 5.** Orthogonal concordance of EPA classifications predicted by DAs and historical in vivo data.

Formulation code	DA-BCOP+	DA-EO+	DA-TTL+	DA-EyeIRR-IS+	Historical in vivo	Majority prediction

A	IV	IV	IV	IV	IV	IV
B	IV	IV	IV	IV	IV	IV
C	IV	IV	IV	IV	IV	IV
D	I	I	I	I	I	I
E	III	III	III	I	I	III
F	I	I	I	I	I	I
G	I	I	I	I	I	I
H	I	I	I	I	I	I
I	I	I	I	I	I	I
J	I	I	I	I	I	I
K	IV	III	III	III	II	III
L	IV	III	III	IV	III	III
M	IV	IV	IV	IV	IV	IV
N	IV	IV	IV	IV	IV	IV
O	IV	III	III	IV	IV	IV
P	IV	IV	IV	IV	IV	IV
Q	II^a^	II	II	II	II	II
R	II	II	I	I	II	II
S	III^a^	III	III	III	III	III
T	III^a^	IV	III	IV	III	III
U	II	II	II	I	II	II
V	I^b^	I^b^	I^b^	I^b^	III	I
W	III	III	III	III	III	III
X	II	II	II	I	II	II
Y	III^a^	III	III	III	II	III
Z	III	IV	IV	IV	III	IV
AA	IV	III	III	III	II	III
AB	II	II	Not tested	Not tested	III	None
AC	III	III	III	IV	III	III
**Orthogonally concordant**	**24/28; 86%**	**26/28; 93%**	**26/28; 93%**	**21/28; 75%**	**22/28; 79%**	
**Orthogonally discordant**	**4/28; 14%**	**2/28; 7%**	**2/28; 7%**	**7/28; 25%**	**6/28; 21%**	
PPE labeling maintained^c^	4	2	2	5	1	
PPE labeling overprotective^c^	0	0	0	2	4	
PPE labeling underprotective^c^	0	0	0	0	1	

a IVIS < 3, but histopathology DoI analysis led to a more severe classification.

b Optional histopathology DoI analysis would lead to a less severe classification (i.e. EPA Cat. II).

c Relative to that of the majority prediction.

Green = orthogonally concordant with majority prediction (i.e. aligned across three or more approaches).

Yellow = orthogonally discordant with majority prediction, but no effect on PPE labeling.

Blue = orthogonally discordant with majority prediction resulting in a change to risk management requirements for PPE (overprotective).

Red = orthogonally discordant with majority prediction resulting in a change to risk management requirements for PPE (underprotective). Specifically, eye goggles are required when using agrochemical formulations labeled EPA Cat. I and II, but they are not required when using those labeled EPA Cat. III and IV.

No fill = no majority alignment across approaches.

## Footnotes

1. Formulations were selected to represent the full range of both EPA and GHS classification systems. Due to differences in classification criteria between the two systems, several formulations that are classified as EPA Cat. III (i.e., mild irritant) are classified as GHS NC (i.e., non-irritant). Thus, while the full range of GHS categories are represented in the test formulations, the number of formulations representing each GHS category are not evenly distributed.

2. All 29 formulations were tested in BCOP (with histopathology), EO, and IVDoI. Only 28 formulations were tested in TTL and EyeIRR-IS because the donated volume of Formulation AB was insufficient for testing in these methods.

3. For the GHS and EPA alignment analyses, classifications based on historical rabbit test data were determined based on the respective in vivo criteria associated with each classification system (Table 1).
