# Supplementary material for: Defined approaches to predict GHS and EPA classifications for ocular irritation potential of agrochemical formulations
Source: Cutan Ocul Toxicol. Author manuscript; Available in PMC 2026 May 15. (PMC12288749; doi:10.1080/15569527.2025.2499552)
Supplement: Supplementary Materials [file NIHMS2086396-supplement-Supplementary_Materials.docx]

Supplemental Table 1. Summary of Testing Results from Stand-alone In Vitro Methods Included in DAs

| **Form. Code** | **BCOP** | | **EO** | **TTL** | | | **EyeIRR-IS** | | | |
| --- | --- | --- | --- | --- | --- | --- | --- | --- | --- | --- |
|  | **IVIS** | **Histo** | **Viability (%)** | **Neat** | **20% dilution** | | **Neat** | | **30% dilution** | |
|  |  |  |  | **Viability, 5 min. (%)** | **Viability, 16 min. (%)** | **Viability, 120 min. (%)** | **LII Run 1** | **LII Run 2** | **LII Run 1** | **LII Run 2** |
| A | 1.2 | Minimal | 93.7 | 75.7 | 98.3 | 57.5 | 4 | 1 | 4 | 1 |
| B | 1.9 | Minimal | 104.6 | 95.1 | 99.4 | 98.1 | 0 | 1 | 0 | 1 |
| C | -0.1 | Minimal | 102.7 | 89.7 | 101.9 | 77.6 | 3 | 3 | 2 | 5 |
| D | 19.7 | Severe | 13.2 | 70 | 85.5 | 1.5 | 12 | 16 | 9 | 6 |
| E | 11 | Mild | 28.5 | 75.9 | 79.9 | 1.8 | 14 | 20 | 14 | 14 |
| F | 39 | Severe | 4.8 | 3.5 | 5.4 | 1.8 | 13 | 17 | 13 | 17 |
| G | 81.9 | Severe | 8.7 | 52.1 | 70 | 2.3 | 12 | 14 | 13 | 16 |
| H | 26.6 | Severe | 5.2 | 2.5 | 3.8 | 1.8 | 20 | 20 | 20 | 20 |
| I | 77.5 | Severe | 5.3 | 6.7 | 81 | 15.5 | 20 | 20 | 13 | 15 |
| J | 15 | Severe | 27.8 | 79 | 74.2 | 2.8 | 20 | 20 | 14 | 14 |
| K | 0 | Minimal | 15.35 | 68.4 | 13.2 | 2.1 | 12 | 12 (Run 3: 13) | 8 | 7 (Run 3: 9) |
| L | 0.6 | Minimal | 22.75 | 77.5 | 82.8 | 10.7 | 9 | 7 | 1 | 6 |
| M | 0.9 | Minimal | 71.1 | 96.8 | 102.4 | 85.4 | 6 | 2 | 2 | 2 |
| N | -0.2 | Minimal | 85.2 | 101.5 | 107 | 90.3 | 6 | 0 | 1 | 0 |
| O | 1.7 | Minimal | 36.6 | 77.7 | 56.6 | 2.4 | 5 | 5 | 5 | 9 |
| P | 1 | Minimal | 101.4 | 101.1 | 96.5 | 79.8 | 2 | 0 | 3 | 1 |
| Q | 1.6 | Moderate | 29.1 | 70 | 18.5 | 2.8 | 13 | 8 (Run 3: 11) | 7 | 5 (Run 3: 6) |
| R | 29.1 | Moderate | 2.2 | 4.7 | 10 | 2 | 12 | 14 | 11 | 12 |
| S | 1.8 | Mild | 26.3 | 66.7 | 18.7 | 2.7 | 20 | 11 (Run 3: 12) | 3 | 6 (Run 3: 8) |
| T | 1.9 | Mild | 78 | 89.4 | 90.6 | 32.3 | 3 | 4 | 0 | 8 |
| U | 23 | Moderate | 9.8 | 55 | 64.9 | 2.5 | 14 | 20 | 14 | 17 |
| V | 59.6 | Moderate | 3.7 | 3.6 | 22.8 | 2.3 | 20 | 20 | 20 | 14 |
| W | 6 | Mild | 33.4 | 85.6 | 22.9 | 2.5 | 12 | 9 (Run 3: 20) | 9 | 6 (Run 3: 1) |
| X | 10.3 | Moderate | 4 | 78.3 | 28.9 | 1.5 | 14 | 14 | 17 | 12 |
| Y | 2.7 | Mild | 19.8 | 83 | 77.5 | 1.7 | 17 | 13 (Run 3: 13) | 5 | 10 (Run 3: 7) |
| Z | 3.9 | Minimal | 91.2 | 94.3 | 101.2 | 93.6 | 4 | 0 | 1 | 0 |
| AA | 2.1 | Minimal | 34.6 | 90.8 | 102.5 | 3.8 | 10 | 14 (Run 3: 17) | 9 | 11 (Run 3: 5) |
| AB | 18.5 | Moderate | 3.5 | Not tested | | | Not tested | | | |
| AC | 3.1 | Mild | 33 | 88.9 | 87.3 | 2.6 | 2 | 3 | 3 | 3 |

Abbreviations: Form. = formulation; histo = histopathology; IVIS = in vitro irritancy score; LII = liquid irritation index.

Supplemental Table 2. Determinants of GHS/EPA Classifications Predicted by DAs

| **Formulation Code** | **DA-BCOP+** | **DA-EO+** | **DA-TTL+** | **DA-EyeIRR-IS+** |
| --- | --- | --- | --- | --- |
| A | BCOP IVIS (histo confirmed) | EO | TTL | EyeIRR-IS |
| B | BCOP IVIS (histo confirmed) | EO | TTL | EyeIRR-IS |
| C | BCOP IVIS (histo confirmed) | EO | TTL | EyeIRR-IS |
| D | BCOP histo | BCOP histo | BCOP histo | BCOP histo |
| E | BCOP histo | BCOP histo | BCOP histo | EyeIRR-IS |
| F | BCOP histo | BCOP histo | TTL | EyeIRR-IS |
| G | BCOP IVIS | BCOP IVIS | BCOP IVIS | EyeIRR-IS |
| H | BCOP histo | BCOP histo | TTL | EyeIRR-IS |
| I | BCOP IVIS | BCOP IVIS | BCOP IVIS | EyeIRR-IS |
| J | BCOP histo | BCOP histo | BCOP histo | EyeIRR-IS |
| K | BCOP IVIS (histo confirmed) | BCOP histo | BCOP histo | BCOP histo |
| L | BCOP IVIS (histo confirmed) | BCOP histo | BCOP histo | EyeIRR-IS |
| M | BCOP IVIS (histo confirmed) | EO | TTL | EyeIRR-IS |
| N | BCOP IVIS (histo confirmed) | EO | TTL | EyeIRR-IS |
| O | BCOP IVIS (histo confirmed) | BCOP histo | BCOP histo | EyeIRR-IS |
| P | BCOP IVIS (histo confirmed) | EO | TTL | EyeIRR-IS |
| Q | BCOP histo | BCOP histo | BCOP histo | BCOP histo |
| R | BCOP histo | BCOP histo | TTL | EyeIRR-IS |
| S | BCOP histo | BCOP histo | BCOP histo | BCOP histo |
| T | BCOP histo | EO | BCOP histo | EyeIRR-IS |
| U | BCOP histo | BCOP histo | BCOP histo | EyeIRR-IS |
| V | BCOP IVIS | BCOP IVIS | TTL | EyeIRR-IS |
| W | BCOP histo | BCOP histo | BCOP histo | BCOP histo |
| X | BCOP histo | BCOP histo | BCOP histo | EyeIRR-IS |
| Y | BCOP histo | BCOP histo | BCOP histo | BCOP histo |
| Z | BCOP histo | EO | TTL | EyeIRR-IS |
| AA | BCOP IVIS (histo confirmed) | BCOP histo | BCOP histo | BCOP histo |
| AB | BCOP histo | BCOP histo | Not tested | Not tested |
| AC | BCOP histo | BCOP histo | BCOP histo | EyeIRR-IS |

Abbreviations: histo = histopathology; IVIS = in vitro irritancy score.

Supplemental Figure 1.:

**
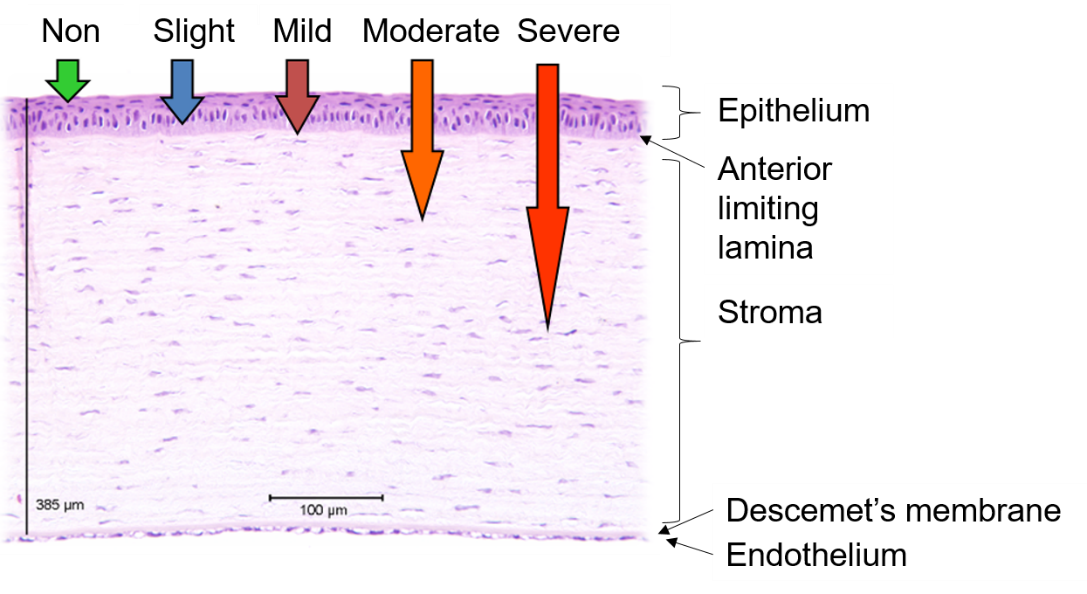
**

Supplemental Figure 1. Histopathological depth of injury prediction model overlaid on a cross section of a rabbit cornea (approximately 400 µm corneal thickness; modified from Scott et al. [2010]). Note that effects described as “minimal” in the current study comprise both “non” and “slight” irritation. Reprinted with permission from van der Zalm et al. (2024) Supplementary Material.

# References

Scott, L., Eskes, C., Hoffmann, S., Adriaens, E., Alepée, N., Bufo, M., Clothier, R., Facchini, D., Faller, C., Guest, R., Harbell, J., Hartung, T., Kamp, H., Varlet, B.L., Meloni, M., McNamee, P., Osborne, R., Pape, W., Pfannenbecker, U., Prinsen, M., Seaman, C., Spielmann, H., Stokes, W., Trouba, K., Berghe, C.V. den, Goethem, F.V., Vassallo, M., Vinardell, P., Zuang, V., 2010. A proposed eye irritation testing strategy to reduce and replace in vivo studies using Bottom-Up and Top-Down approaches. Toxicol. Vitro Int. J. Publ. Assoc. BIBRA 24, 1–9. https://doi.org/10.1016/j.tiv.2009.05.019

van der Zalm, A.J., Daniel, A.B., Raabe, H.A., Choksi, N., Flint Silva, T., Breeden-Alemi, J., O’Dell, L., Kleinstreuer, N.C., Lowit, A.B., Allen, D.G., Clippinger, A.J., 2024. Defined approaches to classify agrochemical formulations into EPA hazard categories developed using EpiOcularTM reconstructed human corneal epithelium and bovine corneal opacity and permeability assays. Cutan. Ocul. Toxicol. 43, 58–68. https://doi.org/10.1080/15569527.2023.2275029
